# Supplementary material for: USP11 controls R-loops by regulating senataxin proteostasis
Source: Nat Commun. 2021 Sep 15;12:5156. doi: 10.1038/s41467-021-25459-w (PMC8443744; doi:10.1038/s41467-021-25459-w)
Supplement: Supplementary file 1 — Supplementary Information [file 41467_2021_25459_MOESM1_ESM.pdf]

Supplementary Figure 1

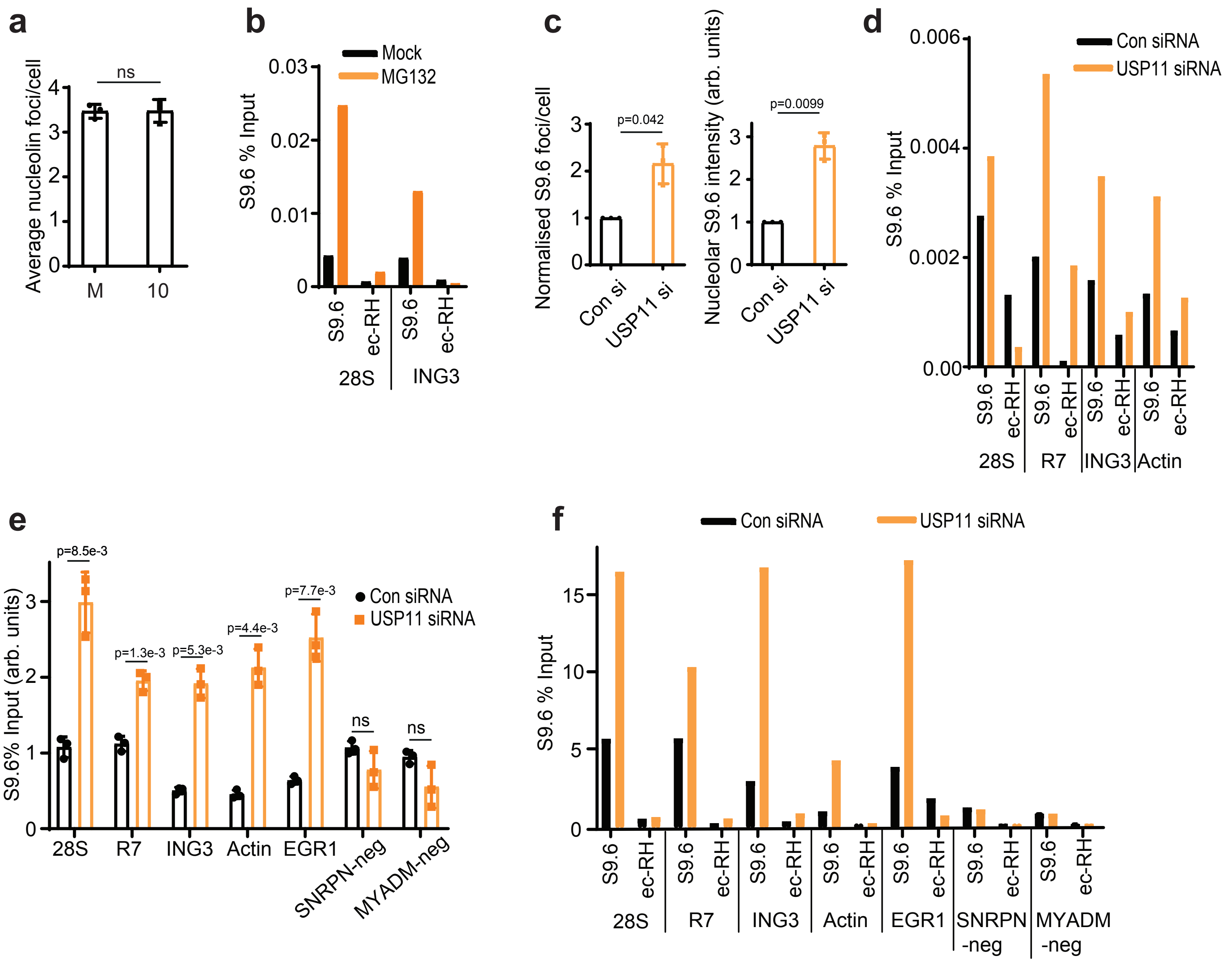

Supplementary Fig. 1. Dysregulation of ubiquitin-proteasome pathway results in increased R-loops.
a) MRC-5 cells were treated with DMSO (M) or 25µM CPT (10) for 10min and immediately harvested for nucleolin immunofluorescence. Data are the average ± SD from 3 biological repeats, each containing at least 100 cells. The average number of nucleoli foci/cell was calculated. ns; p > 0.05, two-tailed Student's t-test.
b) Raw data from a representative repeat of DRIP-qPCR related to Fig. 1f. HEK-293 cells were incubated with mock (DMSO) or 25µM MG132 followed by DNA/RNA immunoprecipitation using S9.6 antibodies. Quantitative PCR was conducted using primers targeting nucleolar (*28S*) and nuclear (*ING3*) loci. *In vitro*, on-bead ec-RNase H (ec-RH) treatment served as a signal validation control.
c) U2-OS cells were transfected with USP11 or Control siRNA (Con si) and examined for R-loops using S9.6/nucleolin immunostaining. Data are the average ± SD from 3 biological repeats, each containing at least 100 cells and presented as average number of S9.6 foci/cell (left panel) and mean S9.6 nucleolar intensity (right panel). Two-tailed Student's t-test.
d) Raw data from a single biological repeat of DRIP-qPCR related to Fig. 2c. Lysates from Control (Con si) and USP11 depleted cells (USP11si) were subjected to DNA/RNA immunoprecipitation (DRIP) using S9.6 antibodies. Quantitative PCR was conducted using primers targeting nucleolar (*28S* and *R7*) and nuclear (*ING3* and *actin*) loci. *In vitro*, on-bead ec-RNase H (ec-RH) treatment served as a signal validation control.
e, f) Lysates from Control (Con siRNA) and USP11 depleted cells (USP11 siRNA) were subjected to a more recently published DNA/RNA immunoprecipitation (DRIP) protocol using S9.6 antibodies[36]. Quantitative PCR was conducted using primers targeting nucleolar (*28S* and *R7*) and nuclear (*ING3, actin, EGR1, SNRPN-neg, MYADM-neg*) loci. *SNRPN-neg* and *MYADM-neg* loci are negative controls. *In vitro*, on-bead ec-RNase H (ec-RH) treatment served as a signal validation control. Pooled repeats (e) and raw % input values from a representative experiment are shown (f), and data represent the average ± SD from 3 biological repeats. ns; p > 0.05, two-tailed Student's t-test.

Supplementary Figure 2

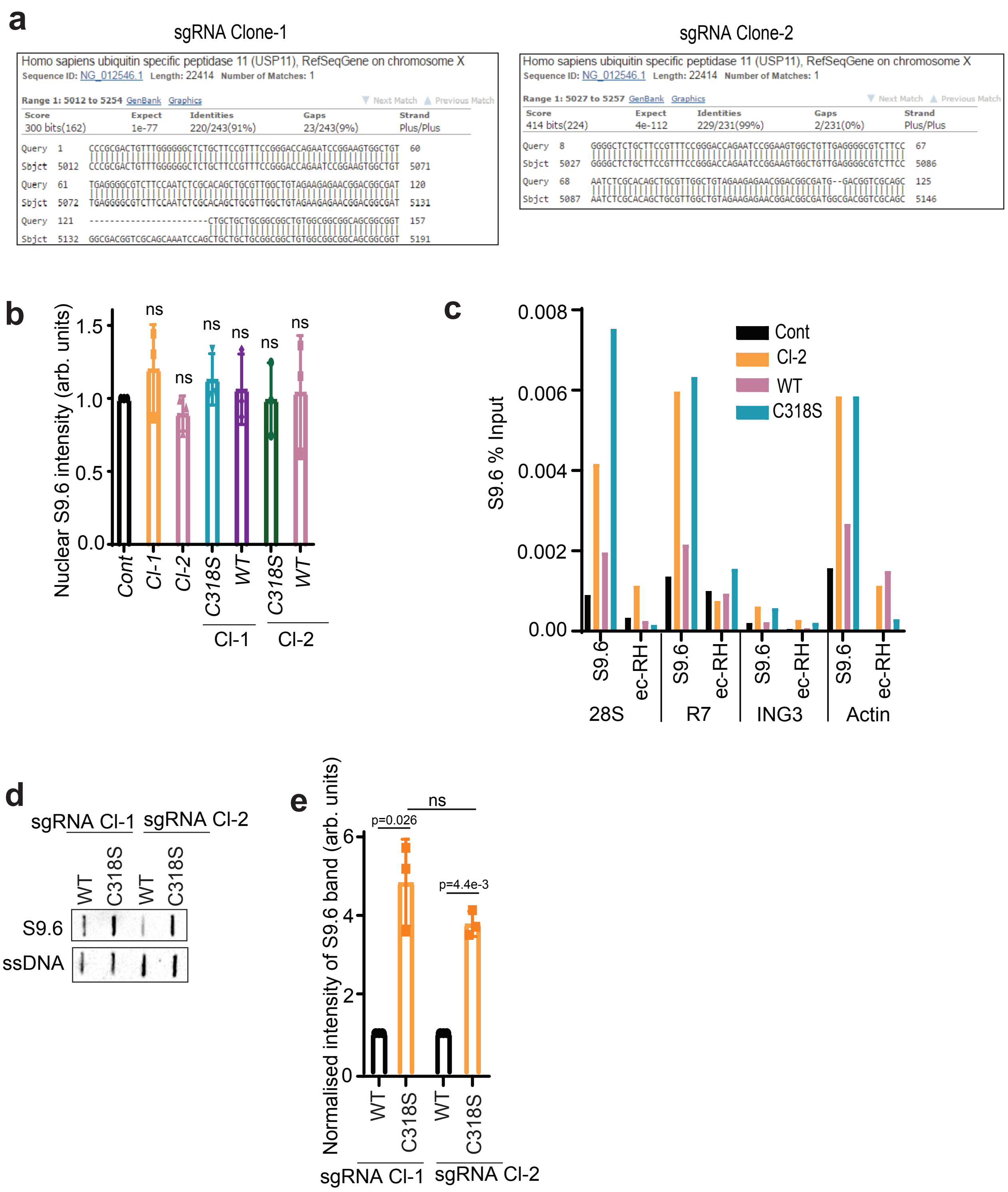

Supplementary Fig. 2 Loss of USP11 triggers R-loop accumulation.

a)Validation of indel mutations in USP11 sgRNA clones. Sanger sequencing reveals a 23-nucleotide deletion in exon 1 of *USP11* gene, Clone-1 (left panel), and a 2-nucleotide deletion in exon 1 of *USP11* gene, Clone-2 (right panel).

b) Corrected total nuclear fluorescence related to Fig. 2f was measured using ImageJ and normalised to control cells. Data are the average ± SD from 3 biological repeats, each containing at least 100 cells. ns; p > 0.05, two-sided Student's t-test.

c) Raw data from a representative experiment of DRIP-qPCR related to Fig. 2g. Lysates from Control HEK-293 (Cont), USP11 sgRNA Cl-2 (Cl-2), USP11-sgRNA Cl-2 complemented with WT USP11 (WT) or C318S USP11 (C318S) were subjected to DNA/RNA immunoprecipitation (DRIP) using S9.6 antibodies. Quantitative PCR was conducted using primers targeting nucleolar (*28S* and *R7*) and nuclear (*ING3* and *actin*) loci. *In vitro*, on-bead ec-RNase H (ec-RH) treatment served as a signal validation control.

d) Lysates from USP11 knockout cells complemented with either wild-type (WT) or catalytically inactive USP11 C318S (C318S) were subjected to a slot blot analysis using S9.6 and α-ssDNA antibodies. A representative experiment of 3 biological repeats is shown. ssDNA signal was used as a loading control.

e) S9.6 band intensities from (d) were normalised to ssDNA and presented as fold enrichment compared to control WT cells. Data are the average of 3 biological repeats and presented as mean ± SD. ns; p>0.05, two-sided Student's t-test.

Supplementary Figure 3

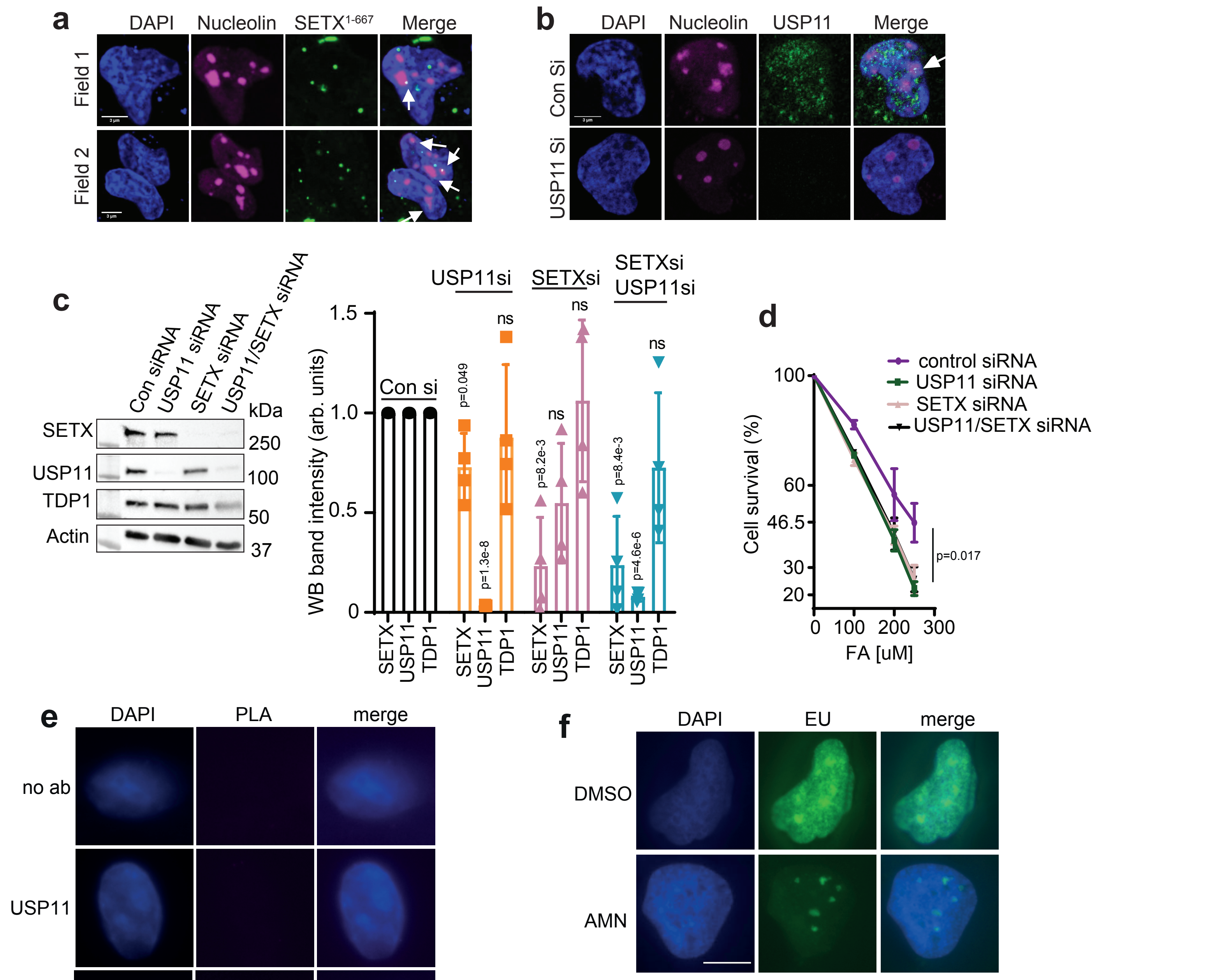

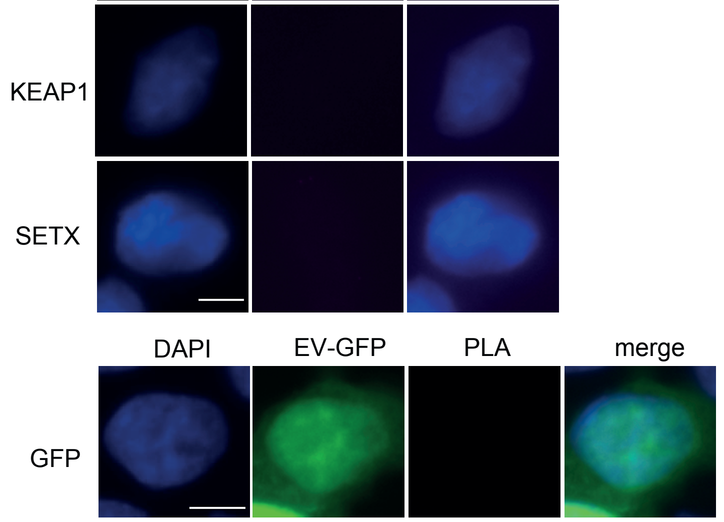

Supplementary Fig. 3 Validation of USP11/SETX knockdown and PLA controls.

a) MRC-5 cells were transfected with eGFP-SETX$^{1-667}$ and examined for nucleolar localisation using anti-nucleolin immunostaining. Representative confocal images from 3 biological repeats are shown, scale bars are 3µm. Arrows depict sites of co-localization between eGFP-SETX$^{1-667}$ and nucleolin.

b) MRC-5 cells were transfected with control (Con Si) or USP11 siRNA and examined for USP11 nucleolar localisation using anti-nucleolin/USP11 immunostaining. Representative confocal images from 3 biological repeats are shown, scale bar is 3µm. The arrow depicts co-localization between USP11 and nucleolin.

c) Lysates from MRC-5 cells treated with Control (Con si), USP11, SETX or USP11 and SETX siRNA were fractionated by SDS-PAGE and analysed by immunoblotting (left). USP11, SETX and TDP1 band intensities were normalised to Actin and presented as fold reduction compared to controls (right). Data are the average of 4 biological repeats and presented as mean ± SD. ns p>0.05, two-sided Student's t-test.

d) MRC-5 cells treated with Control, USP11, SETX or USP11 and SETX siRNA were incubated with the indicated doses of formaldehyde (FA) for 3 hours and left to grow for 7 days. The surviving colonies were counted and % survival calculated relative to mock-treated cells. Data are the mean ± SD from 3 biological repeats. Two-sided Student's t-test

e) Control experiments for proximity ligation assays (PLA) in MRC-5 cells. No antibodies (no ab) or single antibodies as indicated were used. For GFP PLA, MRC-5 cells were transfected with empty vector-eGFP (EV-GFP) first. The scale bar is equal to 10 µm. Images are representative of 3 biological repeats.

f) Validation of alpha-amanitin (AMN) treatment. MRC-5 cells were incubated with 50µM AMN or DMSO over-night, which was followed by 5'-ethynyl uridine (EU) incubation for 1h. After incubation, cells were immediately harvested for EU-staining. The scale bar is equal to 10 µm. Images are representative of 3 biological repeats.

Supplementary Figure 4

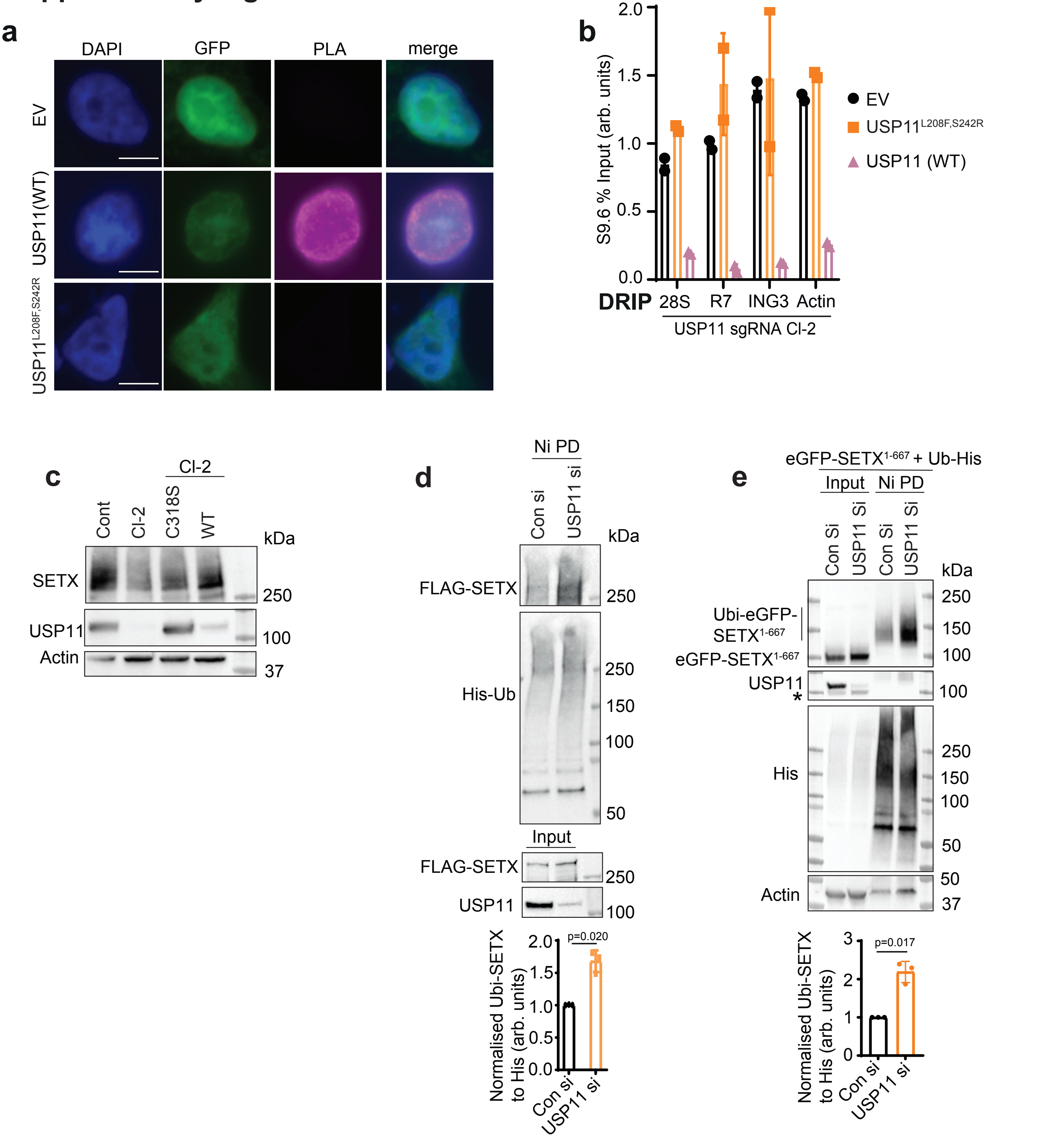

Supplementary Fig.4 USP11^{L208F,S242R}:SETX interaction and ubiquitination of SETX.

a) MRC-5 cells were transcfected with empty vector-eGFP (EV), GFP-USP11 (WT) or GFP-USP11^{L208F,S242R}, and subjected to Proximity Ligation Assay using antibodies against GFP and SETX. Representative images are shown, scale bars are equal to 10µm. Images are representative of 3 biological repeats.

b) Lysates from USP11 sgRNA Cl-2 transiently over-expressing empty vector-eGFP (EV), GFP-USP11^{L208F,S242R} or GFP-USP11 (WT) were subjected to DNA/RNA immunoprecipitation using S9.6 antibodies. Quantitative PCR was conducted using primers targeting nucleolar (*28S* and *R7*) and nuclear (*ING3* and *actin*) loci. The data represent the average ± range from 2 biological repeats.

c) Lysates from parental HEK-293 (Cont), USP11-sgRNA Cl-2 (Cl-2) and USP11-sgRNA Cl-2 complemented with WT USP11 (WT) or active site mutant USP11 (C318S) cells were fractionated by SDS-PAGE and analysed by immunoblotting. Images are representative of 3 biological repeats.

d, e) Control (Con si) and USP11-depleted HEK-293 cells (USP11 si) were transfected with plasmids encoding FLAG-SETX (d) or eGFP-SETX$^{1-667}$ (e) and Ub-His, treated with 10 µM MG132 overnight (proteasomal inhibitor), lysed and subjected to nickel pull-down under denaturing conditions to purify ubiquitinated proteins.Samples were fractionated by SDS-PAGE and analysed by immunoblotting using anti-GFP, FLAG, USP11, His and actin antibodies. *denotes a non-specific band. The band intensities of Ubi-SETX were normalised to His-Ub and presented as fold increase of SETX ubiquitination compared to controls. Data are the average ± SD from 3 biological repeats. p values calculated using two-sided Student's t-test.

Supplementary Figure 5

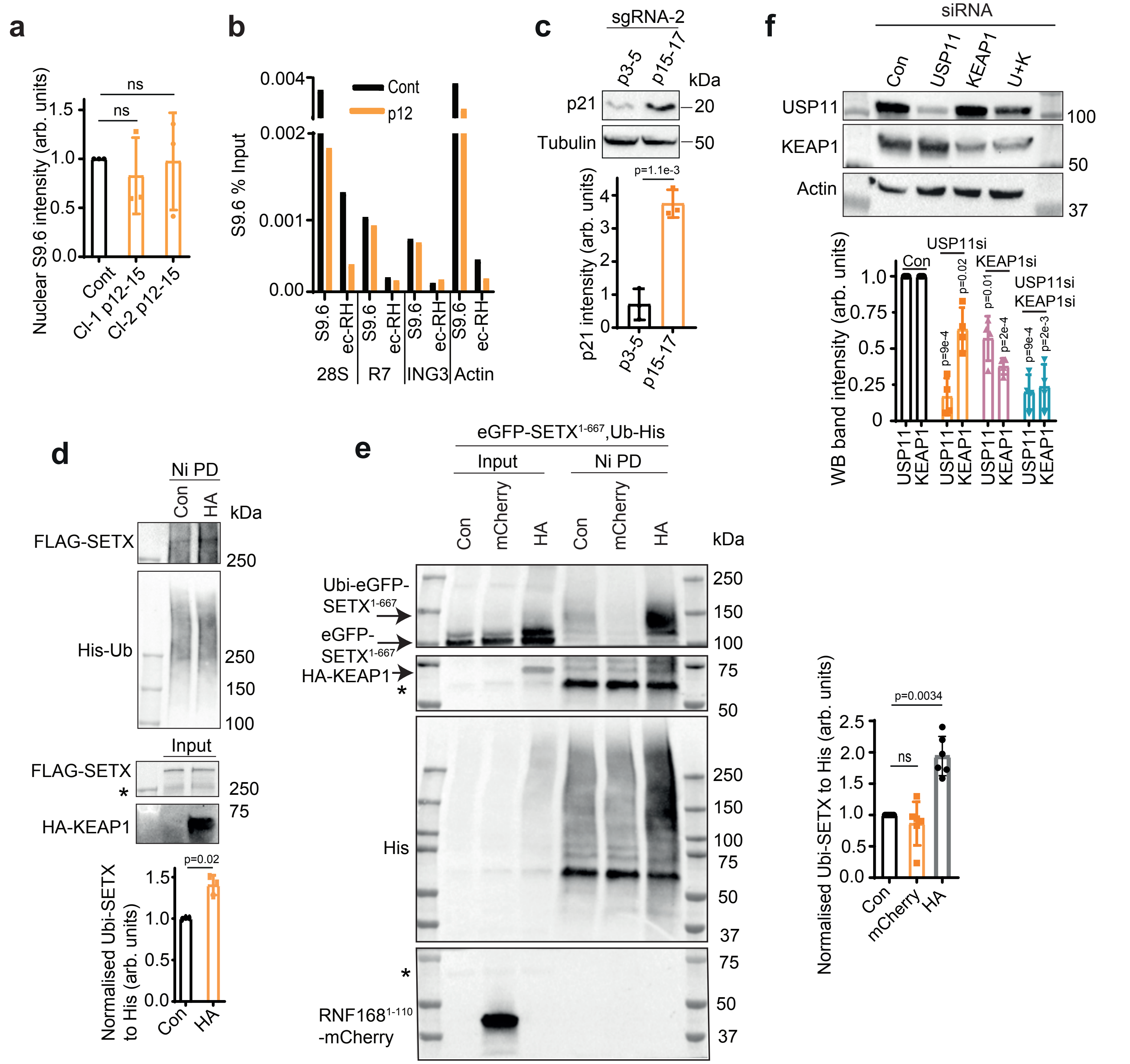

Supplementary Fig. 5. R-loop levels in aged USP11 knockout cells and ubiquitination of SETX.

a) Corrected total nuclear fluorescence of aged USP11 sgRNA Cl-1 and 2 (Cl-1 p12-15; Cl-2 p12-15) related to Fig. 5a was measured using ImageJ and normalised to mock (Cont; HEK-293). Data are the average ± SD from 3 biological repeats, each containing at least 100 cells. ns; $p > 0.05$, two-sided Student t-test.

b) Raw data from a representative experiment of DRIP-qPCR related to Fig 5b. Lysates from Control HEK-293 (Cont) and aged USP11 sgRNA Cl-2 (p12) were subjected to DNA/RNA immunoprecipitation (DRIP) using S9.6 antibodies. Quantitative PCR was conducted using primers targeting nucleolar (*28S* and *R7*) and nuclear (*ING3* and *actin*) loci. *In vitro*, on-bead ec-RNase H (ec-RH) treatment served as a signal validation control.

c) Lysates from young (p3-5) and aged (p15-17) USP11 sgRNA Cl-2 were fractionated by SDS-PAGE and analysed by immunoblotting (top panel). p21 band intensities were normalised to tubulin (bottom panel). Data are the average of 3 biological repeats and presented as mean ± SD. p values were calculated using two-sided Student's t-test.

d, e) Control (Con), RNF168$^{1\text{-}110}$-mCherry (mCherry) and HA-KEAP1 (HA) over-expressing HEK-293 cells were transfected with plasmids encoding Ub-His, FLAG-SETX (e) or eGFP-SETX$^{1\text{-}667}$ (f), lysed and subjected to nickel pull-down under denaturing conditions to purify ubiquitinated proteins. Samples were fractionated by SDS-PAGE and analysed by immunoblotting using anti-GFP, FLAG, USP11, HA, His and mCherry antibodies. *denotes a non-specific band. The band intensities of Ubi-SETX were normalised to His-Ub and presented as fold increase of SETX ubiquitination compared to controls. Data are the average ± SD from 3 biological repeats. ns; $p > 0.05$, two-sided Student's t-test.

f) Lysates from HEK-293 cells treated with scrambled (Con), USP11, KEAP1 or USP11 and KEAP1 (U+K) siRNA were fractionated by SDS-PAGE and analysed by immunoblotting (top panel). USP11 and KEAP1 band intensities were normalised to actin and presented as fold reduction compared to levels in control cells (bottom panel). Data are the average of 4 biological repeats and presented as mean ± SD. p values calculated using two-sided Student's t-test.

Supplementary Figure 6

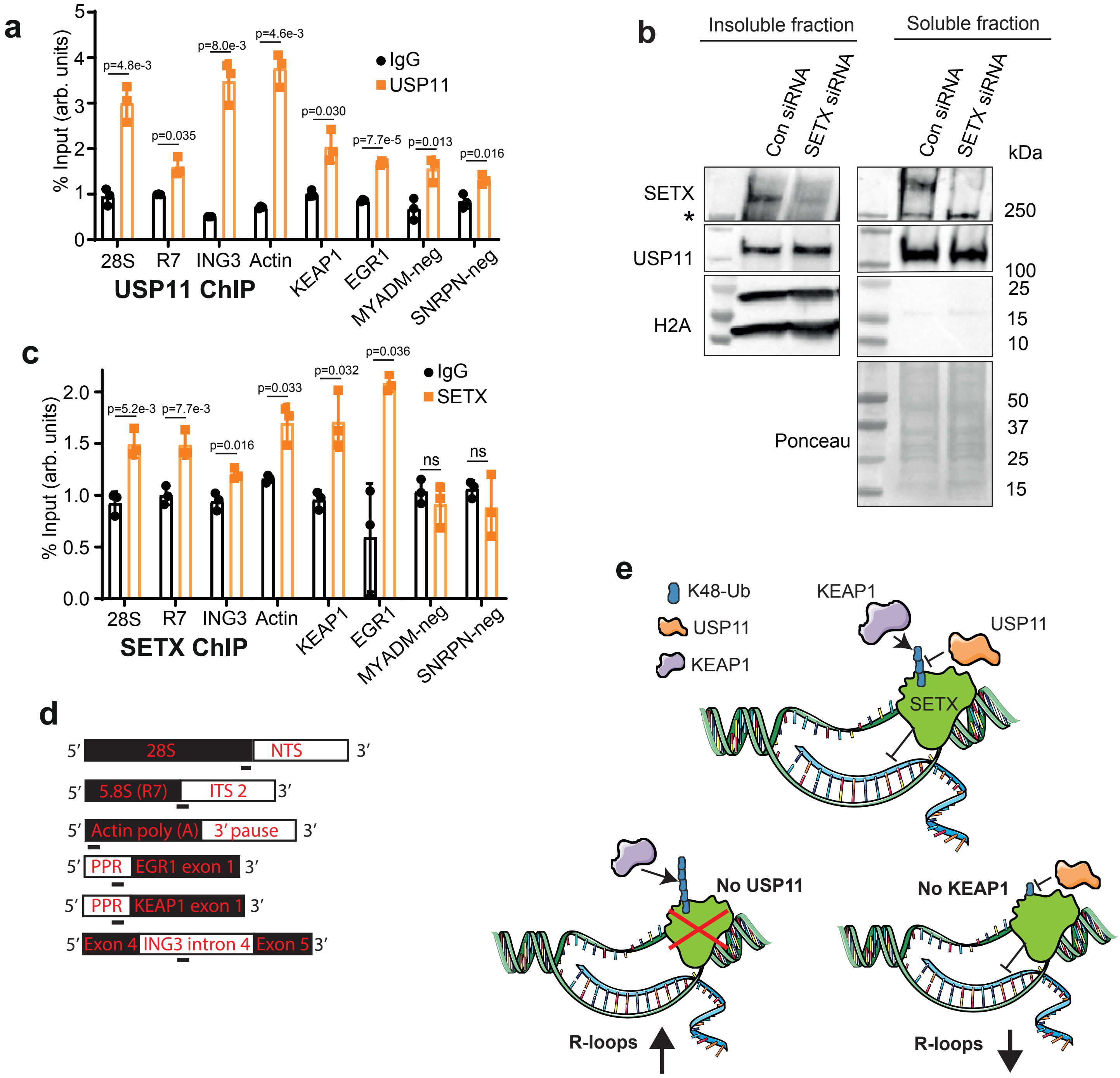

Supplementary Fig. 6. USP11 nuclear fractionation, SETX chromatin binding and a model depciting the role of USP11 and KEAP1.
a) HEK-293 cells were subjected to a USP11 ChIP followed by qPCR using primers targeting nucleolar (*28S, R7*) and nuclear (*ING3, Actin, KEAP1, EGR1, SNRPN-neg, MYADM-neg*) loci. IgG served as a negative control. Data represent the average ± SD from 3 biological repeats. p values calculated using two-sided Student's t-test.
b) HEK-293 cells were transfected with Control siRNA or SETX siRNA and subjected to nuclear fractionation assay followed by immunoblotting. Levels of SETX, USP11 and H2A were analysed. H2A served as a loading control specific to insuluble fraction. * denotes a non-specific band. Images are representative of 3 biological repeats.
c) HEK-293 cells were subjected to a SETX ChIP followed by qPCR using primers targeting nucleolar (*28S, R7*) and nuclear (*ING3, Actin, KEAP1, EGR1, SNRPN-neg, MYADM-neg*) loci. IgG served as a negative control. Data represent the average ± SD from 3 biological repeats. ns; $p > 0.05$, two-sided Student's t-test.
d) A diagram representing PCR amplicons generated by primers used for ChIP and DRIP-qPCR experiments (Fig. 7). NTS - nontranscribed spacer; ITS - intergenic transcribed spacer.
e) KEAP1 and USP11 add and remove K48-Ub chains from SETX respectively. In the absence of USP11, SETX is ubiquitinated and subsequently degraded, which leads to R-loop accumulation. In the absence of KEAP1, SETX is deubiquitinated and thus its protein level is stabilised, which results in R-loop resolution.

Supplementary Table 1. List of primers and DNA sequences used in the study

Gene	NCBI Gene ID	Primer name	Forward Primer (5'-3')	Reverse Primer (5'-3')	Amplicon length	Location	Targeted splice variants	Source	Comment
ACTB	60	Actin β	GCT CAG GGC TTC TTG TCC TT	TCG ATG GGG TAC TTC AGG GT	121bp	Exon 3	All	Li,M., Pokharel,S., Wang,J.T., Xu,X. and Liu,Y. (2015) RECQ5-dependent SUMOylation of DNA topoisomerase I prevents transcription-associated genome instability. *Nat. Commun.*, 6, 1–13.	Reference gene for RT-qPCR
USP11	8237	USP11	TGGAAGGCGAGGATTATGTGC	ATGACCTTGCGTTCAATGGGT	102bp	Exon 4	All	Zhou,Z., Luo,A., Shrivastava,I., He,M., Huang,Y., Bahar,I., Liu,Z. and Wan,Y. (2017) Regulation of XIAP Turnover Reveals a Role for USP11 in Promotion of Tumorigenesis. *EBioMedicine*, 15, 48–61.	Target gene
SETX	23064	SETX	CTT CAT CCT CGG ACA TTT GAG	TTA ATA ATG GCA CCA CGC TTC	176bp	Exon 26	All	Skourti-Stathaki,K., Proudfoot,N.J. and Gromak,N. (2011) Human senataxin resolves RNA/DNA hybrids formed at transcriptional pause sites to promote Xrn2-dependent termination. *Mol. Cell*, 42, 794–805.	Target gene
KEAP1	9817	KEAP1 (RT-qPCR)	TGG CCA AGC AAG AGG AGT TC	GGC TGA TGA GGG TCA CCA GTT	60bp	Exon 3	All	Hussong,M., Börno,S.T., Kerick,M., Wunderlich,A., Franz,A., Sültmann,H., Timmermann,B., Lehrach,H., Hirsch-Kauffmann,M. and Schweiger,M.R. (2014) The bromodomain protein BRD4 regulates the KEAP1/NRF2-dependent oxidative stress response. *Cell Death Dis.*, 5, 1–11.	Target gene for RT-qPCR
KEAP1	9817	KEAP1 (ChIP/DRIP-qPCR)	GGA AAG GAG CGG CGA TTC	CAT CCA GCA ACG AAA TCG GG	137	Promoter proximal region	All	This study	Target gene for ChIP/DRIP-qPCR
RNA28SN3	109910382	28S	CAG GGG AAT CCG ACT GTT TA	AT GAC GAG GCA TTT GGC TAC	173bp	ribosomal RNA	All	Johnston,R., D'Costa,Z., Ray,S., Gorski,J., Paul Harkin,D., Mullan,P. and Panov,K.I. (2016) The identification of a novel role for BRCA1 in regulating RNA polymerase I transcription. *Oncotarget*, 7, 68097–68110.	Target gene
RNA5-8SN3	109910381	R7	GAC ACT TCG AAC GCA CTT G	CTC AGA CAG GCG TAG CCC CG	55bp	ribosomal RNA	All	Shen,W., Sun,H., De Hoyos,C.L., Bailey,J.K., Liang,X.H. and Crooke,S.T. (2017) Dynamic nucleoplasmic and nucleolar localization of mammalian RNase H1 in response to RNAP I transcriptional R-loops. *Nucleic Acids Res.*, 45, 10672–10692.	Target gene
ING3	54556	ING3	TTT TTC TTC TCT AAC TAC CCT CCC C	GTG CCC TAA TCT GAA TGA CTA CA	99bp	intron 4	All	Halász,L., Karányi,Z., Boros-oláh,B., Kuik-rózsa,T., Sipos,É., Nagy,É., Mosolygó-l,Á., Mázló,A., Rajnavölgyi,É., Halmos,G., *et al.* (2017) RNA-DNA hybrid (R-loop) immunoprecipitation mapping: an analytical workflow to evaluate inherent biases. 10.1101/gr.219394.116.6.	Target gene

ACTB	60	Actin	TTA CCC AGA GTG CAG GTG TG	CCC CAA TAA GCA GGA ACA GA	104bp	5' of 3' pause site	All	Skourti-Stathaki,K., Proudfoot,N.J. and Gromak,N. (2011) Human senataxin resolves RNA/DNA hybrids formed at transcriptional pause sites to promote Xrn2-dependent termination. *Mol. Cell*, 42, 794–805.	Target gene
EGR1	1958	EGR1	CATAGGGAAG CCCCTCTTTC	CTTGTGGTGAGGG GTCACTT	90bp	Promoter proximal region	All	Ginno,P., Lott,P., Christensen,H., Korf,I. and Chédin,F. (2012) R-loop formation is a distinctive characteristic of unmethylated human CpG island promoters. *Mol. Cell*, 45, 814–825.	Target gene
FRA3B	2272	FRA3B	TTAGCCTACTT CAGGGTTTCT	TGGAGAGGTTACT ACTGGCA	148bp	Common Fragile Site	All	Björkman, A., Johansen, S.L., Lin, L., Schertzer, M., Kanellis, D.C., Katsori, A.-M., Christensen, S.T., Luo, Y., Andersen, J.S., Elsässer, S.J., et al. (2020). Human RTEL1 associates with Poldip3 to facilitate responses to replication stress and R-loop resolution. Genes Dev. 34, 1065–1074.	Target gene
FRA16D	2463	FRA16	CAGCCAGCAC TCCTTCTCAA	CTCTGTGGAGAAG CCAAGCA	149bp	Common Fragile Site	All	Björkman, A., Johansen, S.L., Lin, L., Schertzer, M., Kanellis, D.C., Katsori, A.-M., Christensen, S.T., Luo, Y., Andersen, J.S., Elsässer, S.J., et al. (2020). Human RTEL1 associates with Poldip3 to facilitate responses to replication stress and R-loop resolution. Genes Dev. 34, 1065–1074.	Target gene
SNRPN	6638	SNRPN-neg	GCCAAATGAG TGAGGATGGT	TCCTCTCTGCCTG ACTCCAT	102bp	5' UTR	All	Ginno,P., Lott,P., Christensen,H., Korf,I. and Chédin,F. (2012) R-loop formation is a distinctive characteristic of unmethylated human CpG island promoters. *Mol. Cell*, 45, 814–825.	Target gene
MYADM	91663	MYADM-neg	TGCATCTACAT CCGCAAAAG	AGAGTGGACGCT GCAGAAAT	146bp	5' UTR	All	Ginno,P., Lott,P., Christensen,H., Korf,I. and Chédin,F. (2012) R-loop formation is a distinctive characteristic of unmethylated human CpG island promoters. *Mol. Cell*, 45, 814–825.	Target gene

SDM & Cloning primers

Name of the primer	Forward Primer (5'-3')	Reverse Primer (5'-3')	Method	Purpose	Template plasmid
USP11 (C318S)	CAA TCT GGG CAA CAC GAG CTT CAT GAA CTC GGC	GCC GAG TTC ATG AAG CTC GTG TTG CCC AGA TTG	SDM	Generation of FLAG-HA-USP11(C318S)	FLAG-HA-USP11
SETX(1-667)	ACA AGT CCG GAC TCA GAT CTA TGA GCA CAT GTT GTT GG	5'CAC TCA CTG GTA CTG GCC CTT TAG TCA CCT TCT ATA GTG TTA TC	Gibson Cloning	To amplify SETX(1-667) for eGFP-SETX(1-667)	pCMV6-XL-SETX
peGFP-C1	AGG GCC AGT ACC AGT GAG	AGA TCT GAG TCC GGA CTT G	Gibson Cloning	To amplify peGFP-C1 for eGFP-SETX(1-667)	peGFP-C1
USP11 (WT)	CAA GTC CGG ACT CAG ATC TAT GGC AGT AGC CCC GCG A	ACT CAC TGG TAC TGG CCC TTC AAT TAA CAT CCA TGA ACT CAG AGC TGG	Gibson Cloning	To amplify USP11 for eGFP-USP11(WT)	FLAG-HA-USP11
peGFP-C1	AGG GCC AGT ACC AGT GAG	AGA TCT GAG TCC GGA CTT G	Gibson Cloning	To amplify peGFP-C1 for eGFP-USP11 (WT)	peGFP-C1
RNF168(1-110)	TCAGATCTCGAGCTCAAGCTATGGCTCTACC CAAAGAC	ATGTCGACTGCAGAATTCGAAGGTTTACTGAGC AGACG	Gibson Cloning	To amplify RNF168(1-110) for pRNF168(1-110)-mCherry	peGFP-RNF168

pmCherry-N1	TCGAATTCTGCAGTCGAC	AGCTTGAGCTCGAGATCTG	Gibson Cloning	To amplify pmCherry-N1 for pRNF168(1-110)-mCherry	pmCherry-N1
USP11 (L208F)	CCAAGAACTCAGAAGGCTCTTTCGATAGGTTGTATGACACACAC	GTGTGTGTCATACAACCTATCGAAAGAGCCTTCTGAGTTCTTGG	SDM	Generation of FLAG-HA-USP11(L208F)	FLAG-HA-USP11
USP11 (S242R)	GGCACTTGGCCCAGAGCACAGCTGCATGTC	GACATGCAGCTGTGCTCTGGGCCAAGTGCC	SDM	Generation of FLAG-HA-USP11(L208F, S242R)	FLAG-HA-USP11(L208F)

Supplementary Table 2. List of siRNA and gRNA used in this study

siRNA	siRNA Sequence
siControl	5’ UAA UGU AUU GGA ACG GAU 3’
siUSP11 #1	5’ GCG CAC AGC UGC AUG UCA U 3’
siUSP11 #2	5’ GAG AAG CAC UGG UAU AAG C 3’
siUSP11 #3	5’ GGA CCG UGA UGA UAU CUU C 3’
siUSP11 #4	5’ GAA GAA GCG UUA CUA UGA C 3’
siSETX #1	5’ GCA CGU CAG UCA UGC GUA A 3’
siSETX #2	5’ GCA AUA AGC UCA UCC UAG U 3’
siSETX #3	5’ GCU CAA CUC UCC AAA UAG A 3’
siSETX #4	5’ UAG CAC AGG UUG UUA AUC A 3’
siKEAP1	5’ CAG CAG AAC UGU ACC UGU U 3’

gRNA	gRNA Sequence
USP11_Exon1 top strand	5’ CAC CGA GAA CGG ACG GCG ATG GCG A 3’
USP11_Exon 1 bottom strand	5’ AAA CTC GCC ATC GCC GTC CGT TCT C 3’

Supplementary Table 3. List of antibodies used in this study

Antibody	Dilution / Quantity	Purpose	Source	Identifier
TDP1	1:1000	IB	Abcam	ab4166
Beta-actin	1:2000	IB	Sungene Biotech	KM9001
S9.6	1:500	IF	Kerafast	ENH001
	20 or 28 µg	DRIP		
USP11	1:1000	PLA	Bethyl	A301-613A
	1:2000	IB/IF		
	2 µg	ChIP		
K48 ubiquitin	1:1000	IB	Abcam	ab140601
GFP	1:1000	PLA	Abcam	ab290
	1:2000	IB/IF		
SETX	1:200	PLA	Abnova	2579-2676
	5 µg	ChIP	Bethyl	A301-104A
	1:1000	IB		
His	1:1000	IB	Abcam	ab18184
HA	1:1000	IB	Santa Cruz	F-7
GAPDH	1:2000	IB	Calbiotem	CB1001
Beta-tubulin	1:2000	IB	Abcam	ab7792
RPA-194	1:1000	IB	Santa Cruz	sc-48385
Cyclin A2	1:1000	IB	Cell Signalling	#4656
Nucleolin	1:1000	IB/IF	Abcam	ab136649, ab22758
mCherry	1:1000	IB	Abcam	ab167453
KEAP1	1:1000	WB/PLA	Abcam	ab139729
yH2A.X	2 µg	ChIP	EMD Millipore	05-636
XPF	1 µg	ChIP	Abcam	ab76948
p21	1:200	WB	Invitrogen	14-6715-81
RNA Pol II	1:1000	IB	Santa Cruz	sc-55492
ssDNA	1:1000	Slot blot	EMD Millipore	#MAB3868
Alexa Fluor 555 goat anti-rabbit IgG	1:500	IF	Thermo Fisher	A21428
Alexa Fluor 594 goat anti-rabbit IgG	1:500	IF	Thermo Fisher	A110012
Alexa Fluor 594 goat anti-mouse IgG	1:500	IF	Thermo Fisher	A11005
Alexa Fluor 488 goat anti-rabbit IgG	1:1000	IF	Thermo Fisher	A11008
Rabbit IgG	2 µg	IP	Invitrogen	02-6102
Goat Anti-Rabbit IgG (H+L)-HRP Conjugate	1:4000	IB	Bio-Rad Laboratories	170-6515
Goat Anti-Mouse IgG (H+L)-HRP Conjugate	1:4000	IB	Bio-Rad Laboratories	170-6516

Supplementary Table 4. List of plasmids used in this study:

Plasmid	Purpose	Reference / Source
HA-KEAP1	Transient recombinant protein expression	Plasmid # 21556 - Addgene
FLAG-HA-USP11	Complementation of USP11 sgRNA clones, SDM, cloning	Plasmid # 22566 - Addgene
FLAG-HA-USP11^{C318S}	Complementation of USP11 sgRNA clones	This study
pCMV6-XL-SETX	Cloning	Plasmid SC308907 – OriGene
peGFP-SETX$^{1-667}$	Pull-down	This study
FLAG-SETX	Pull-down	Gift from S. Wilson
peGFP-C1	Cloning, Pull-down, PLA	Clonetech
peGFP-USP11	Transient recombinant protein expression, PLA, SDM	This study
peGFP-USP11$^{L208F,\ S242R}$	Transient recombinant protein expression, PLA	This study
RNF168(1-110)-mCherry	Pull-down	Gift from S.El-Khamisy
Ubiquitin-10xHis	Pull-down	Gift from S.El-Khamisy
pSpCas9(BB)-2A-GFP_PX458	Gene silencing	Plasmid # 48138 - Addgene
