# Supplementary material for: USP11 controls R-loops by regulating senataxin proteostasis
Source: Nat Commun. 2021 Sep 15;12:5156. doi: 10.1038/s41467-021-25459-w (PMC8443744; doi:10.1038/s41467-021-25459-w)
Supplement: Supplementary file 3 — Description of Additional Supplementary Files [file 41467_2021_25459_MOESM3_ESM.pdf]

Description of Additional Supplementary Files

File Name: Supplementary Data 1

Description: Supplementary Data 1. Raw DUB siRNA screen data. MRC-5 cells were reverse transfected on 96-well plates containing siRNA for 99 human deubiquitylases, mock-treated or treated with 25 µM camptothecin (CPT) for 10 min and immunostained with S9.6 antibodies. The S9.6 foci/cell values were quantified by Custom Module of MetaXpress® software and normalised to siRNA controls. Data were analysed by two-tailed student's t-test.
