# Supplementary material for: USP11 controls R-loops by regulating senataxin proteostasis
Source: Nat Commun. 2021 Sep 15;12:5156. doi: 10.1038/s41467-021-25459-w (PMC8443744; doi:10.1038/s41467-021-25459-w)
Supplement: Supplementary file 5 — Reporting Summary [file 41467_2021_25459_MOESM5_ESM.pdf]

nature portfolio

Corresponding author(s): Sherif El-Khamisy

Last updated by author(s): 09/07/2021

Reporting Summary

Nature Portfolio wishes to improve the reproducibility of the work that we publish. This form provides structure for consistency and transparency in reporting. For further information on Nature Portfolio policies, see our Editorial Policies and the Editorial Policy Checklist.

Statistics

For all statistical analyses, confirm that the following items are present in the figure legend, table legend, main text, or Methods section.

n/a	Confirmed	
☐	☒	The exact sample size (*n*) for each experimental group/condition, given as a discrete number and unit of measurement
☐	☒	A statement on whether measurements were taken from distinct samples or whether the same sample was measured repeatedly
☐	☒	The statistical test(s) used AND whether they are one- or two-sided<br>*Only common tests should be described solely by name; describe more complex techniques in the Methods section.*
☒	☐	A description of all covariates tested
☒	☐	A description of any assumptions or corrections, such as tests of normality and adjustment for multiple comparisons
☐	☒	A full description of the statistical parameters including central tendency (e.g. means) or other basic estimates (e.g. regression coefficient) AND variation (e.g. standard deviation) or associated estimates of uncertainty (e.g. confidence intervals)
☐	☒	For null hypothesis testing, the test statistic (e.g. *F*, *t*, *r*) with confidence intervals, effect sizes, degrees of freedom and *P* value noted<br>*Give P values as exact values whenever suitable.*
☒	☐	For Bayesian analysis, information on the choice of priors and Markov chain Monte Carlo settings
☒	☐	For hierarchical and complex designs, identification of the appropriate level for tests and full reporting of outcomes
☒	☐	Estimates of effect sizes (e.g. Cohen's *d*, Pearson's *r*), indicating how they were calculated

Our web collection on statistics for biologists contains articles on many of the points above.

Software and code

Policy information about availability of computer code

Data collection: Image Express Micro XLS Widefield High Content Analysis System (Molecular Devices, San Jose, CA) was used to collect DUB siRNA screen images. Rotor Gene 6000 series Software 1.7 (Corbett Life Science) was used to determine the quantification cycle (Cq) for qPCR and RT-qPCR experiments.

Data analysis: Custom Module of MetaXpress Software version 6 (Molecular Devices, San Jose, CA) was used to process DUB siRNA screen images. Image J version 1.53k was used to analyse immunofluorescent images.

For manuscripts utilizing custom algorithms or software that are central to the research but not yet described in published literature, software must be made available to editors and reviewers. We strongly encourage code deposition in a community repository (e.g. GitHub). See the Nature Portfolio guidelines for submitting code & software for further information.

Data

Policy information about availability of data

All manuscripts must include a data availability statement. This statement should provide the following information, where applicable:

- Accession codes, unique identifiers, or web links for publicly available datasets
- A description of any restrictions on data availability
- For clinical datasets or third party data, please ensure that the statement adheres to our policy

Source data for the DUB siRNA screen and all of the raw images of western/slot blots are provided with the paper.

Field-specific reporting

Please select the one below that is the best fit for your research. If you are not sure, read the appropriate sections before making your selection.

☒ Life sciences ☐ Behavioural & social sciences ☐ Ecological, evolutionary & environmental sciences

For a reference copy of the document with all sections, see nature.com/documents/nr-reporting-summary-flat.pdf

Life sciences study design

All studies must disclose on these points even when the disclosure is negative.

Sample size	Chosen sample size was sufficient for a robust statistical calculation. All statistical calculations were derived from at least 3 biological repeats. For microscopy based-asssays, each biological repeat included sufficient cell numbers and the n value was equal to or higher than 3 in order to ensure high degree of statistical significance.
Data exclusions	No data was excluded
Replication	Experiments were performed multiples times (three or more) with multiple biological repeats (usually three). All attempts at replication were successful.
Randomization	Allocation of samples was random.
Blinding	Investigators were double blinded to the sample allocation during data allocation and analysis.

Reporting for specific materials, systems and methods

We require information from authors about some types of materials, experimental systems and methods used in many studies. Here, indicate whether each material, system or method listed is relevant to your study. If you are not sure if a list item applies to your research, read the appropriate section before selecting a response.

Materials & experimental systems

n/a	Involved in the study	
☐	☒	Antibodies
☐	☒	Eukaryotic cell lines
☒	☐	Palaeontology and archaeology
☒	☐	Animals and other organisms
☒	☐	Human research participants
☒	☐	Clinical data
☒	☐	Dual use research of concern

Methods

n/a	Involved in the study	
☒	☐	ChIP-seq
☒	☐	Flow cytometry
☒	☐	MRI-based neuroimaging

Antibodies

Antibodies used	All antibodies are listed in Supplementary Table 3 with detailed description.
Validation	All antibodies were validated by suppliers and also have been validated byother publications. In addition, the key antibodies were validated by siRNA knockdown, in vitro ec-RNase H treatment or plasmid over-expression - for instance see Supplementary Figures 1d, 3a, 5f.

Eukaryotic cell lines

Policy information about cell lines

Cell line source(s)	HEK-293 (ATCC)<br>MRC-5 (ATCC)<br>U2-OS (ATCC)
Authentication	Authenticated by imaging procedure.
Mycoplasma contamination	All cell lines came negative for Mycoplasma contamination.
Commonly misidentified lines<br>(See ICLAC register)	None were used.
